# Oncological safety and fertility outcomes of controlled ovarian stimulation in patients with early-stage endometrial cancer

**DOI:** 10.1016/j.xfre.2025.07.008

**Published:** 2025-07-24

**Authors:** Stavroula L. Kastora, Iliana Armata, Dhivya Chandrasekaran, Radha Graham, Nicola MacDonald, Ephia Yasmin

**Affiliations:** a Women's Health Division, Reproductive Medicine Unit, University College London Hospital, London, United Kingdom; b Department of Obstetrics and Gynaecology, Peterborough City Hospital, Edith Cavell Campus, Bretton Gate, Peterborough, United Kingdom

**Keywords:** Early-stage endometrioid cancer, fertility preservation, pregnancy outcomes, controlled ovarian stimulation

## Abstract

**Objective:**

To study the oncological safety and efficacy with regard to reproductive outcomes of controlled ovarian stimulation (OS) with levonorgestrel-releasing intrauterine system (LNG-IUS).

**Design:**

Retrospective cohort study.

**Subjects:**

Patients presenting with complex endometrial hyperplasia or early-stage endometrial carcinoma (EC), managed with levonorgestrel-releasing intrauterine system.

**Exposure:**

Controlled OS.

**Main Outcome Measures:**

The primary outcome of the present study was to evaluate the oncological safety of controlled OS on disease progression, and the secondary outcome was to explore fertility outcomes in patients treated with LNG-IUS. Time-to-event outcomes were visualized as Kaplan-Meier plots and hazard ratios (HRs) with 95% confidence intervals (CIs).

**Results:**

Thirty-four patients, with a median age of 34 years (interquartile range [IQR], 30–38) and body mass index of 29.6 kg/m^2^ (IQR, 26–36.9), were included in our analysis. Of the patients, 76.5% were diagnosed with grade 1 EC, and 73.5% sought fertility counseling. The median anti-mullerian hormone level was 22.40 pmol/L (IQR, 8.7–55.9). Eleven patients underwent OS with a median peak estradiol level of 1,406 pmol/L (IQR, 694.0–9,577), median of 5 (IQR, 2–17) oocytes retrieved, and median of 4 (IQR, 1–6) embryos stored. There was no significant difference between patients who did and those who did not undergo OS in terms of recurrence (HR, 1.16 [95% CI, 0.36–3.76]) or disease upgrading (HR, 0.71 [95% CI, 0.13–3.97]) for the first 50 months after LNG-IUS placement. Eleven out of 34 (32.35%) patients attempted to conceive during the follow-up period; of those, 54% achieved a live birth (3 by spontaneous conception, 1 by assisted reproductive technology, and 2 by surrogacy with own embryos).

**Conclusion:**

There may be an oncologically safe window of opportunity for fertility preservation and conception for patients on LNG-IUS diagnosed with early-stage EC, wishing to complete their family.

Endometrial carcinoma (EC) is the fourth most common malignant tumor in women across the United Kingdom (1). Complex endometrial hyperplasia with atypia (CAH) is a known precursor of endometrioid EC. The current gold-standard treatment is hysterectomy with or without bilateral salpingectomy (2). However, 5% of women with early-stage EC and CAH are younger than 40 years old, and such approach has terminal effects on their fertility. Recent evidence has demonstrated the oncological safety of oral progestins and levonorgestrel-releasing intrauterine system (LNG-IUS) as a fertility-sparing alternative (3). Most of the available evidence is based on retrospective studies limited by a with a short 6-month follow-up period, which, in turn, obscures oncological safety outcomes (4, 5). Furthermore, the effectiveness of oral progestins as an adjunct to LNG-IUS in achieving remission remains uncertain. Lastly, the safety of ovarian stimulation (OS), in view of follow-up hysteroscopy findings, in women undergoing fertility-sparing management of early-stage EC remains underinvestigated. Fertility-sparing options may be temporary, and as such, a window of opportunity to cryopreserve oocytes or embryos needs to be grasped. This may reduce the time to pregnancy when histologic normality is achieved through medical management. Additionally, such intervention can offer the option of surrogacy if response to medical treatment is suboptimal necessitating definitive management in the form of hysterectomy and bilateral salpingo-oophorectomy (BSO) (6, 7). Here, we evaluated the oncological safety of LNG-IUS with or without oral progestins for early-stage EC/CAH, explored fertility outcomes in the LNG-IUS treated population, and highlighted the effects of OS on follow-up hysteroscopic histopathology and recurrence rate.

## Materials and methods

This retrospective observational study included patients presenting with CAH/early endometrioid cancer at University College London Hospitals (Gynaecology Oncology Department), managed with LNG-IUS with or without oral progestins between the years of 2014 and 2024. Patients aged 18–45 years with confirmed CAH or grade 1–2 endometrioid EC managed conservatively with LNG-IUS ± oral progestins were included in the present study. Patients with grade 3 disease, nonendometrioid histology, or prior hysterectomy or those with <6 months of follow-up were excluded. Patient demographic (age, body mass index [BMI], and parity) and clinical variables (histopathology report at referral, date of LNG-IUS placement, and subsequent hysteroscopy pathology reports [diagnosis and date]) were recorded for each patient. Fertility-specific parameters were also recorded, specifically fertility referral (binary outcome), controlled OS (COS) (binary outcome), anti-mullerian hormone (AMH; pmol/L), baseline estradiol (pmol/L), peak estradiol (pmol/L) for those who underwent OS, the number of oocytes retrieved/embryos stored, and live birth (binary outcome). Institutional review board approval was sought and deemed not required given the retrospective and anonymized nature of the patient data.

### COS protocol

All patients who underwent controlled ovarian hyperstimulation had documented disease regression or normal histology on endometrial biopsy within 3 months before stimulation. Controlled OS performed with human menopausal gonadotropin and recombinant follicle-stimulating hormone, guided by AMH and antral follicle count. Levonorgestrel-releasing intrauterine system remained in situ. Medroxyprogesterone acetate used from day 4 (progestin-primed OS). Trigger with human chorionic gonadotropin or gonadotropin-releasing hormone agonist depending on luteinizing hormone suppression.

### Oncological follow-up

Endometrial biopsy is performed every 3 months if there is active disease or therapy change initiated, biopsies are conducted every 6 months if there is no progression of disease or signs of regression and annually when 2 negative biopsies (complete regression) are achieved.

### Outcomes

The primary outcomes included time to recurrence, upgrading of disease on subsequent hysteroscopy, or final hysterectomy specimen. The secondary outcomes included fertility outcomes, namely, oocytes retrieved, number of embryos stored, and subsequent live birth. Additionally, patients who underwent COS were subgrouped, and oncological safety outcomes were compared between those and the remaining cohort.

### Statistical analysis

Continuous variables were reported as medians and interquartile ranges (IQRs). Categorical variables were numerically encoded to facilitate downstream. Statistical difference of continuous variables was assessed by the Mann-Whitney *U* test. Time-to-event outcomes were visualized as Kaplan-Meier curves, effect sizes were reported as hazard ratios (HRs) (Mantel-Haenszel) with 95% confidence intervals (CIs), and statistical significance was assessed through the Mantel-Cox test (*P* value). The statistical significance cutoff was set at *P*<.05. GraphPad Prism V. 10 (academic license) was employed for statistical analysis and graph generation.

## Results

**Figure 1:**
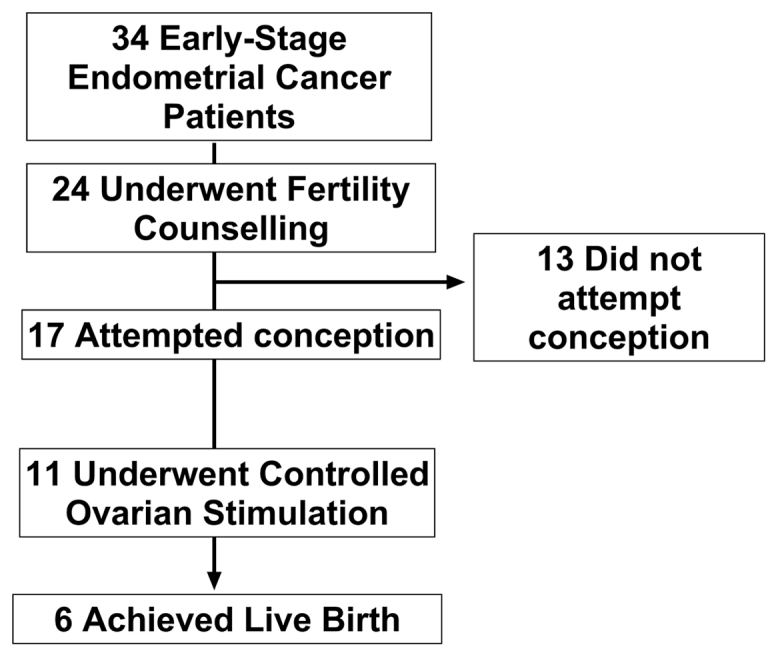
Flowchart illustrating patient distribution and outcomes in the cohort of 34 women with early-stage endometrial cancer managed with levonorgestrel-releasing intrauterine system ± oral progestins.

A total of 34 patients with a median age of 34 years (IQR, 30–38) and BMI of 29.6 kg/m^2^ (IQR, 26–36.9) were included in our analysis (Fig. 1, Supplemental Fig. 1A and B, available online, and Table 1). Twenty-six patients (76.5%) were diagnosed with grade 1 EC, of whom 22 (64.71%) were on adjunct oral progestins (Supplemental Fig. 1C). Patients were followed up for a median of 4 years (range, 2–9) with 91.2% remaining under consultant follow-up at present. Three patients (8.6%) were identified with mismatch repair deficiency suggestive of Lynch syndrome. Notably, 41.17% of patients had a diagnosis of polycystic ovary syndrome (PCOS). A total of 25 patients (73.5%) sought fertility counseling (Supplemental Fig. 2A). At first encounter with the fertility services, the median AMH level was 22.40 pmol/L (IQR, 8.7–55.9) (Supplemental Fig. 2B and C), and the median estradiol level was 239.0 pmol/L (IQR, 176.5–425.0) (N = 13) (Supplemental Fig. 2D). Eleven patients underwent a single cycle of OS with a median peak estradiol level of 1,406 pmol/L (IQR, 694.0–9,577) (Supplemental Fig. 2D). From these patients, a median of 5 oocytes were retrieved (IQR, 2–17) with a median of 4 embryos (IQR, 1–6) frozen (Fig. 1).

**Table 1:** Patient variables: demographic and clinical cohort characteristics.

Demographics, N = 34	Frequency
Age (y)	34 (IQR, 30–38)
BMI (kg/m^2^)	29.6 (IQR, 26–36.9)
**Past medical history**	
Lynch syndrome (N, %)	3 (8.6%)
Polycystic ovary syndrome (N, %)	14 (41.17%)
**Oncology parameters**	
Diagnosis (N, %)	Complex hyperplasia with atypia: 5 (14.71%) Grade 1 EC: 26 (76.47%) Grade 2 EC: 3 (8.82%)
Myoinvasion (N, %)	0 (0%)
MMR status (N, %)	Retained: 31 (91.17%) Loss: 3 (8.6%)
**Treatment**	
Adjunct oral progestogens (N, %)	22 (64.71%)
**Fertility parameters, N = 11**	
Parity before treatment	0 (range, 0–1)
Parity after treatment	0 (range, 0–2)
Anti-mullerian hormone, pmol/L	22.40 (IQR, 8.7–55.9)
No. of oocytes retrieved	5 (IQR, 2–17)
No. of MII oocytes retrieved	4 (IQR, 0–20)
Embryos frozen	4 (IQR, 1–6)
Baseline estradiol (pmol/L)	239.0 (IQR, 176.5–425.0)
Peak estradiol (pmol/L)	1,406 pmol/L

*Note:* Values displayed as medians and interquartile ranges, 25th to 75th centiles, unless otherwise specified. BMI = body mass index; EC = endometrial carcinoma; IQR = interquartile range; MII = metaphase II; MMR = mismatch repair.

**Figure 2:**
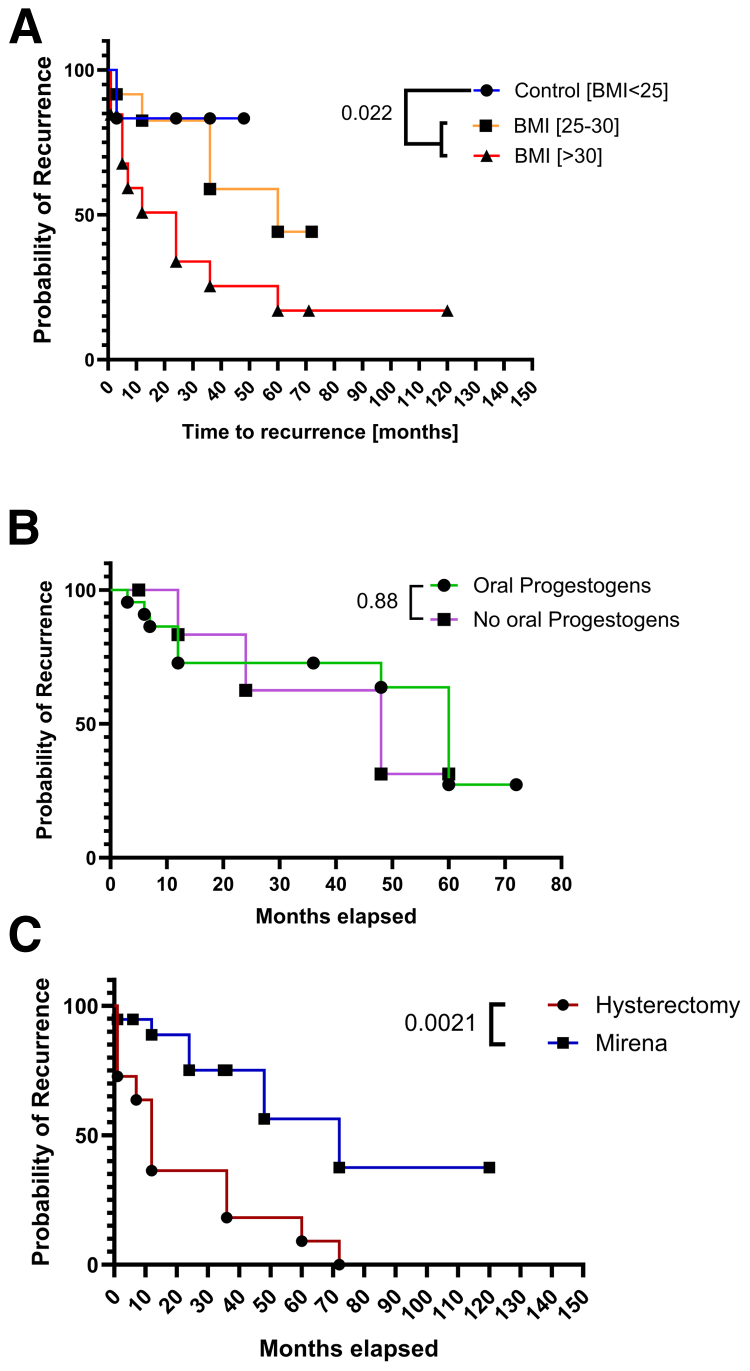
Oncological variables. Recurrence events as binary outcomes. (**A**) Time to recurrence (body mass index [BMI] of <25 kg/m^2^ vs. BMI of >30 kg/m^2^) (*P*=.022). (**B**) Time to recurrence (on adjunct oral progestogens vs. levonorgestrel-releasing intrauterine system [LNG-IUS] only) (*P*=.8934). (**C**) Time to recurrence (patients who eventually had total laparoscopic hysterectomy vs. those on continuing LNG-IUS) (*P*=.0021).

In terms of overall oncological safety, time to recurrence was related strongly to increased BMI. Specifically, patients with a lower BMI (<25 kg/m^2^) were likely to have a recurrence much later (50 months) compared with women with a BMI between 25 and 30 kg/m^2^ (HR, 1.81 [95% CI, 0.26–12.66]; *P*=.55) and those with a higher BMI (>30 kg/m^2^) (HR, 3.51 [95% CI, 0.94–13.09]; *P*=.02) who experienced disease recurrence much earlier, 38 months and 10 months, respectively (Fig. 2A). There was no significant difference noted between the patients who were on adjunct oral progestins and the ones who were not (HR, 0.90 [95% CI, 0.2171–3.747]; *P*=.89) (Fig. 2B). Patients who eventually underwent surgical management were those who experienced recurrence earlier, between 10–20 months and 48–72 months, after placement of LNG-IUS (HR, 5.511 [95% CI, 1.86–16.34]; *P*=.0021) (Fig. 2C).

**Figure 3:**
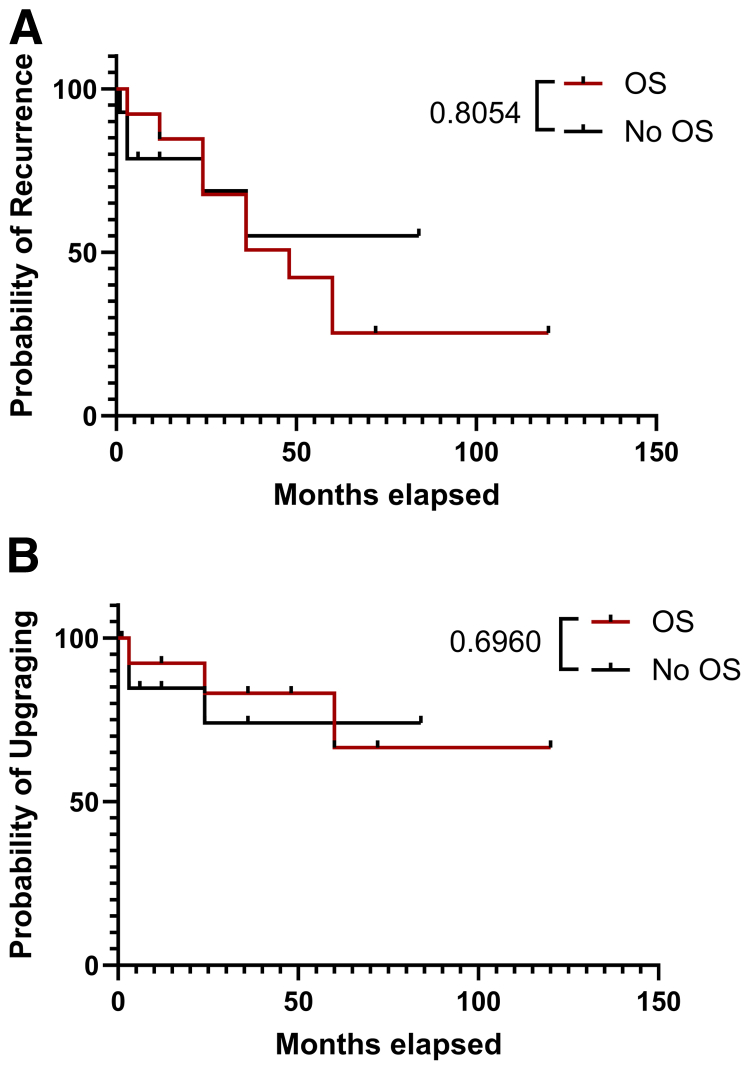
Kaplan-Meier survival of patients exposed to ovarian stimulation (OS) vs. no OS. (**A**) Recurrence events (hazard ratio, 1.159 [95% confidence interval, 0.3575–3.761]; *P*=.8553). (**B**) Upgrading events (hazard ratio, 0.7092 [95% confidence interval, 0.1266–3.974]; *P*=. 5746). For all events analyses, time 0 was the time of levonorgestrel-releasing intrauterine system placement.

Eleven patients (32.35%), with a median age of 35 years (IQR, 30–40) and BMI of 26 kg/m^2^ (IQR, 25–34), attempted to conceive during the follow-up period (Supplemental Fig. 2D). A total of 6 (54%) of 11 achieved a live birth, three by spontaneous conception, one via assisted reproduction techniques (in vitro fertilization/intracytoplasmic sperm injection), and two by surrogacy with own embryos. Parity before treatment was of a median of 0 (range, 0–1) vs. a median of 0 (range, 0–2) after COS and fertility preservation. There was no statistically significant difference noted between patients who did and those who did not undergo COS in terms of recurrence (unadjusted HR, 1.16 [95% CI, 0.36–3.76]; *P*=.80) (Fig. 3A and Supplemental Table 1, available online) or disease upgrading at subsequent hysteroscopy (unadjusted HR, 0.71 [95% CI, 0.13–3.97]; *P*=.57) for the first 50 months after LNG-IUS placement (Fig. 3B and Supplemental Table 1). Additionally, adjustment for age at diagnosis, BMI, preexisting PCOS diagnosis, and time since diagnosis (in years) did not reveal a statistically significant risk of either recurrence (adjusted HR, 1.47 [95% CI, 0.42–5.19]; *P*=.55) or disease upgrading at subsequent hysteroscopy (adjusted HR, 0.31 [95% CI, 0.04–2.75]; *P*=.29) (Supplemental Table 2). Overall, this study suggests that a single cycle of COS with LNG-IUS in situ for local disease control appears to be an oncologically safe option for women who wish to maintain fertility and subsequently attempt to conceive.

## Discussion

The present retrospective study of 34 patients with early endometrioid EC suggests that LNG-IUS appears to be a safe fertility-sparing alternative toward the management of early-stage EC. In terms of oncological safety, adjunct progestins do not appear to delay time to recurrence, whereas a BMI of >30 kg/m^2^ appears to be a poor prognostic factor in terms of recurrence. A live pregnancy rate of 54% was achieved, with no significant difference between patients who did and those who did not undergo COS in terms of recurrence or disease upgrading at subsequent hysteroscopy for the first 50 months after LNG-IUS placement. Patients with overall lower BMI (<25 kg/m^2^) may have a wider, recurrence-free window of opportunity for natural conception, and therefore, weight loss should be strongly supported especially for patients with PCOS or other metabolic syndromes, However, it is crucial to mention that the overall recurrence rate was still notably high, regardless of COS (47%), and therefore, the need for definitive treatment remains crucial in women who have completed their family. Another notable observation, relevant to both EC risk and fertility preservation outcomes, was the high prevalence of PCOS within our cohort. Although the AMH levels were consistently elevated, the median number of mature oocytes retrieved remained modest. This paradox is likely attributable to the underlying PCOS phenotype, present in 41.17% of patients, where an increased AMH level reflects increased antral follicle count but is frequently accompanied by impaired follicular synchrony and reduced oocyte maturity (8, 9). Moreover, the use of the progestin-primed protocol may be beneficial in the stimulation of women who require progesterone for endometrial protection (10). Close follow-up, personalized to each patient’s baseline risk factors with multidisciplinary team input from the oncology and fertility teams, that monitors treatment response and progression of symptoms or disease is vital.

The gold-standard treatment for early-stage EC in medically operable women involves a hysterectomy, typically accompanied by a BSO. However, for younger women, BSO is suboptimal because it induces surgical menopause, leading to long-term estrogen deprivation and associated adverse effects, including cognitive decline, urogenital atrophy, and skeletal deterioration (11). In cases where surgery alone is insufficient for disease control, adjuvant therapies such as brachytherapy are employed (12). The LNG-IUS, commonly known as Mirena, is a widely used form of long-acting reversible contraception that is also indicated for the treatment of abnormal uterine bleeding, including menorrhagia. Levonorgestrel exerts its therapeutic effect by suppressing endometrial proliferation, inducing endometrial atrophy through decidualization and suppression of endometrial gland activity (13). Recently, LNG-IUS has gained attention as a potential alternative therapy for hyperplasia and early-stage EC, particularly in women deemed inoperable. Comparative studies assessing the efficacy of systemic progestin therapy vs. LNG-IUS for the treatment of hyperplasia have demonstrated that LNG-IUS therapy yields higher regression rates and lower hysterectomy rates than oral progestogen treatments (14). Systemic progestogen therapy, such as medroxyprogesterone acetate, is effective in treating hormone-sensitive hyperplasia and tumors (15). However, the down-regulation of progesterone receptors limits its therapeutic duration (16). Additionally, systemic therapy is often associated with poor patient compliance due to adverse effects, including nausea, weight gain, abnormal vaginal bleeding, and an increased risk of breast cancer (17). Despite these challenges, systemic progestins have been used alongside LNG-IUS in efforts to preserve fertility while maintaining disease control.

Although the use of LNG-IUS for the treatment of endometrial hyperplasia and early-stage EC shows promise and has been endorsed by the National Comprehensive Cancer Network (18), its efficacy as a definitive treatment remains under investigation. Some women exhibit resistance to LNG-IUS treatment for hyperplasia and early-stage EC, with the underlying mechanisms of this recalcitrance poorly understood. Surgical intervention remains associated with high cure rates and low morbidity, particularly in early-stage EC (19). Therefore, studies have focused on the identification of predictive biomarkers that could refine patient selection for LNG-IUS therapy, minimizing the risk of disease progression and broadening its use to women desiring to retain their fertility, on a predicted response (6, 7, 20).

After fertility-sparing treatment (FST), the reported pregnancy rates range from approximately 18% to 34%, and in turn, pregnancy should be encouraged as soon as possible (21). Higher pregnancy rates have been observed in women using LNG-IUDs (56%), oral progestins alone (52.1%), and hysteroscopic focal resection combined with progestins (47.8%) (22, 23). In women who underwent assisted reproductive technology, the pregnancy and live birth rates were between 50% and 75% (24). Of note, the recurrence rate after FSTs is relatively high, with an estimated rate of approximately 25%, increasing over time rendering long-term follow-up essential (25). The continuous administration of oral progestins and/or the use of LNG-IUDs after FSTs as well as childbirth has been shown to significantly improve prognosis (26). Increased estrogen levels during ovulation induction are considered a potential risk factor for EC progression or recurrence. However, the use of letrozole in conjunction with gonadotropins during COS has been suggested to prevent excessive estrogen levels (4). Notably, assisted reproductive technology has not been associated with an increased recurrence of disease (5). Furthermore, previous retrospective studies have indicated that LNG-IUDs do not impair OS cycle outcomes (5). In fact, clinical pregnancy and live birth rates appear unaffected by the presence of LNG-IUDs (27, 28). However, the impact of COS on oncological outcomes in patients with EC remains obscure. Emerging evidence suggest that patients who underwent COS with LNG-IUS in situ had lower recurrence rates during COS than those without LNG-IUS in situ (12.1% vs. 35.5%, *P*=.027), and the clinical pregnancy (42.4% vs. 52.9%, *P*=.392) and live birth (21.2% vs. 29.4%, *P*=.444) rates were found to be comparable between the LNG-IUD and control groups (29). Importantly, the use of LNG-IUD during COS assessed in 67 Chinese women with early-stage EC or complex hyperplasia with atypia was found to be a favorable factor for lower recurrence rates after COS (HR, 0.263; 95 % CI, 0.084–0.822; *P*=.022) (29). Consistent with the study by Yin et al. (29), our data suggest no statistically significant difference between patients who underwent COS and those who did not, especially for the first 50 months of LNG-IUS placement. The latter suggests a window of opportunity for COS and conception for patients who wish to conceive. The certainty on which this window may be offered may be augmented, in future, by predictive markers of response.

### Strengths and implications for future research

Despite limited by the small sample size and its retrospective nature, our study presents among the largest case series examining controlled ovarian hyperstimulation safety in early-stage EC managed with LNG-IUS. Our data suggest that there is an oncologically safe window of opportunity for fertility preservation and conception for patients diagnosed with early-stage EC, wishing to complete their family. Additionally, the ethnic diversity of the reviewed population alleviates genetic and ethnic bottlenecks, which may impact other data sets (29) and may overall affect COS response and/or recurrence rate. However, given the limited sample size, adjusted analysis for ethnic background on recurrence effect sizes was not deemed possible and presents a significant opportunity for future research. With that in mind, further studies are required to supplement the current evidence availability and support clear recommendations regarding fertility preservation in patients diagnosed with early-stage EC.

## Conclusion

There may be an oncologically safe window of opportunity for fertility preservation and conception for patients on LNG-IUS diagnosed with early-stage EC, wishing to complete their family. Frequent and close input from both the fertility and oncology teams is necessary. However, further studies are required to augment aggregate sample size to reach robust evidence supporting COS with LNG-IUS in terms of oncological and fertility outcomes.

## CRediT Authorship Contribution Statement

**Stavroula L. Kastora:** Writing – review & editing, Writing – original draft, Visualization, Methodology, Investigation, Formal analysis, Data curation. **Iliana Armata:** Writing – review & editing, Writing – original draft. **Dhivya Chandrasekaran:** Writing – review & editing, Supervision, Conceptualization. **Radha Graham:** Writing – review & editing, Data curation. **Nicola MacDonald:** Writing – review & editing, Supervision. **Ephia Yasmin:** Writing – review & editing, Supervision, Conceptualization.

## Declaration of Interests

S.L.K. has nothing to disclose. I.A. has nothing to disclose. D.C. has nothing to disclose. R.G. has nothing to disclose. N.M. has nothing to disclose. E.Y. has nothing to disclose.
