# Supplementary material for: Oncological safety and fertility outcomes of controlled ovarian stimulation in patients with early-stage endometrial cancer
Source: F S Rep. 2025 Jul 24;6(3):335–40. doi: 10.1016/j.xfre.2025.07.008 (PMC12496423; doi:10.1016/j.xfre.2025.07.008)
Supplement: Supplemental Table 1 [file mmc1.docx]

**Article Title:** **Oncological safety and fertility outcomes of controlled ovarian stimulation in early-stage endometrial cancer patients.**

| Variable | COS [N=11] | No COS [N=23] | P-value |
| --- | --- | --- | --- |
| Age (Years) | 35 [IQR 30, 40] | 33 [IQR 30, 38] | 0.4945 |
| BMI (kg/m^2^) | 26 [IQR 25, 34] | 32.11 [IQR 29, 38.2] | 0.1783 |
| Time since diagnosis (Years) | 4.08 [IQR 2, 3] | 3.70 [IQR 3, 5] | 0.3603 |
| Recurrence event (Binary outcome)  Population affected | 1. [IQR 0, 1]   8; [69.2%] | 0.00 [IQR 0, 1]  9; [38.1%] | 0.0858  0.157 |

**Table S1.** Comparison of baseline characteristics between patients who underwent controlled ovarian stimulation (COS) and those who did not. Values are presented as median and interquartile range (IQR). P-values were calculated using the Mann-Whitney U test.
