# Supplementary material for: Oncological safety and fertility outcomes of controlled ovarian stimulation in patients with early-stage endometrial cancer
Source: F S Rep. 2025 Jul 24;6(3):335–40. doi: 10.1016/j.xfre.2025.07.008 (PMC12496423; doi:10.1016/j.xfre.2025.07.008)
Supplement: Supplemental Table 2 [file mmc2.docx]

**Article Title:** **Oncological safety and fertility outcomes of controlled ovarian stimulation in early-stage endometrial cancer patients.**

| **Outcome** | **Recurrence** | | | **Disease upgrading at subsequent hysteroscopy** | | |
| --- | --- | --- | --- | --- | --- | --- |
| **Variable** | **HR** | **95% CI (Lower–Upper)** | **P-value** | **HR (adjusted)** | **95% CI (Lower–Upper)** | **P-value** |
| COS (vs No COS) | 1.47 | 0.42 – 5.19 | 0.55 | 0.31 | 0.04 – 2.75 | 0.29 |
| Age (years) | 1.05 | 0.96 – 1.16 | 0.29 | 1.06 | 0.89 – 1.27 | 0.51 |
| BMI (kg/m²) | 1.02 | 0.97 – 1.07 | 0.49 | 1.02 | 0.95 – 1.08 | 0.65 |
| Time since diagnosis (years) | 1.02 | 0.83 – 1.25 | 0.88 | 1.2 | 0.87 – 1.65 | 0.26 |
| PCOS | 1.47 | 0.45 – 4.85 | 0.52 | 3.3 | 0.56 – 19.31 | 0.19 |

**Table S2. Adjusted Hazard Ratio for Recurrence and Disease upgrading at subsequent hysteroscopy by** Age (years), BMI (kg/m²) and Time since diagnosis (years). Poisson regression approximation for Cox modelling.
