# Supplementary material for: Oncological safety and fertility outcomes of controlled ovarian stimulation in patients with early-stage endometrial cancer
Source: F S Rep. 2025 Jul 24;6(3):335–40. doi: 10.1016/j.xfre.2025.07.008 (PMC12496423; doi:10.1016/j.xfre.2025.07.008)
Supplement: Supplemental Figures [file mmc3.docx]

**Article Title: Oncological safety and fertility outcomes of controlled ovarian stimulation in early-stage endometrial cancer patients.**

**Supplementary Figures 1 and 2**

**Figure S1.** Patient demographics (Age, BMI) (A), Diagnosis at first encounter, 3 cases of Mismatch repair (MMR) loss [8.57%] (B) and % [N] of population on add-on oral progestogens (C).Í

**Figure. S2** Fertility-related variables of population. (A) Referral to fertility team upon encounter (B) Median AMH on first encounter (C) Random vs. peak oestradiol (depicted only for those that underwent ovarian stimulation); Mann-Whitney test (D) Attempt to conceive whilst on treatment. Statistical significance (P value) displayed in numerical fashion.
